# Supplementary material for: From sequence to enzyme mechanism using multi-label machine learning
Source: BMC Bioinformatics. 2014 May 19;15:150. doi: 10.1186/1471-2105-15-150 (PMC4229970; doi:10.1186/1471-2105-15-150)
Supplement: Additional file 2 — Java code of ml2db. Additional file ml2db_code.tar.gz contains the Java source code to run the multi-label machine learning experiments and save the results to database. The code’s Javadoc is included. [file 1471-2105-15-150-S2.zip › additional file 2/ml2db/ml2db/doc/mulan/core/class-use/MulanRuntimeException.html]

Uses of Class mulan.core.MulanRuntimeException


JavaScript is disabled on your browser.


- Overview
- Package
- Class
- Use
- Tree
- Deprecated
- Index
- Help

- Prev
- Next

- Frames
- No Frames

- All Classes

## Uses of Class mulan.core.MulanRuntimeException

- Packages that use MulanRuntimeException

  | Package | Description |
  | --- | --- |
  |  |  |
  | --- | --- |
  | mulan.classifier |  |
  | mulan.core |  |
  | mulan.data |  |
- - ### Uses of MulanRuntimeException in mulan.classifier

    Subclasses of MulanRuntimeException in mulan.classifier

    | Modifier and Type | Class and Description |
    | --- | --- |
    |  |  |
    | --- | --- |
    | `class` | `InvalidDataException` Exception thrown by the `MultiLabelLearner` when presented with invalid data or data which can not be processed by the specific learner type. |
    | `class` | `LearnerException` Represents a root base class for exceptions thrown by learners. |
    | `class` | `ModelInitializationException` Exception thrown by the `MultiLabelLearner` when learner is queried for prediction on data before model is internally built by learning from training data. |
  - ### Uses of MulanRuntimeException in mulan.core

    Subclasses of MulanRuntimeException in mulan.core

    | Modifier and Type | Class and Description |
    | --- | --- |
    |  |  |
    | --- | --- |
    | `class` | `ArgumentNullException` This exception is raised when a null reference is passed to a method that does not accept a null reference for an argument. |
    | `class` | `WekaException` The convenience exception, which can be used to wrap up checked general `Exception` commonly thrown by underlying Weka library into anonymous runtime exception. |
  - ### Uses of MulanRuntimeException in mulan.data

    Subclasses of MulanRuntimeException in mulan.data

    | Modifier and Type | Class and Description |
    | --- | --- |
    |  |  |
    | --- | --- |
    | `class` | `DataLoadException` The exception is thrown to indicate an error while loading the data. |

- Overview
- Package
- Class
- Use
- Tree
- Deprecated
- Index
- Help

- Prev
- Next

- Frames
- No Frames

- All Classes
